# Genome-wide identification and characterization of the soybean SOD family during alkaline stress

**DOI:** 10.7717/peerj.8457

**Published:** 2020-02-05

**Authors:** Wenxiu Lu, Huizi Duanmu, Yanhua Qiao, Xiaoxia Jin, Yang Yu, Lijie Yu, Chao Chen

**Affiliations:** 1 School of Life Science and Technology, Harbin Normal University, Harbin, China; 2 Key Laboratory of Molecular Biology, College of Heilongjiang Province, College of Life Sciences, Heilongjiang University, Harbin, China; 3 Key Laboratory of Soybean Molecular Design Breeding, Northeast Institute of Geography and Agroecology, Chinese Academy of Sciences, Harbin, China

**Keywords:** Soybean, Phylogenetic analysis, Evolution, Alkaline, Expression patterns

## Abstract

**Background:**

Superoxide dismutase (SOD) proteins, as one kind of the antioxidant enzymes, play critical roles in plant response to various environment stresses. Even though its functions in the oxidative stress were very well characterized, the roles of *SOD* family genes in regulating alkaline stress response are not fully reported.

**Methods:**

We identified the potential family members by using Hidden Markov model and soybean genome database. The neighbor-joining phylogenetic tree and exon-intron structures were generated by using software MEGA 5.0 and GSDS online server, respectively. Furthermore, the conserved motifs were analyzed by MEME online server. The syntenic analysis was conducted using Circos-0.69. Additionally, the expression levels of soybean *SOD* genes under alkaline stress were identified by qRT-PCR.

**Results:**

In this study, we identified 13 potential *SOD* genes in soybean genome. Phylogenetic analysis suggested that *SOD* genes could be classified into three subfamilies, including MnSODs (*GmMSD1–2*), FeSODs (*GmFSD1–5*) and Cu/ZnSODs (*GmCSD1–6*). We further investigated the gene structure, chromosomal locations and gene-duplication, conserved domains and promoter *cis*-elements of the soybean *SOD* genes. We also explored the expression profiles of soybean *SOD* genes in different tissues and alkaline, salt and cold stresses, based on the transcriptome data. In addition, we detected their expression patterns in roots and leaves by qRT-PCR under alkaline stress, and found that different *SOD* subfamily genes may play different roles in response to alkaline stress. These results also confirmed the hypothesis that the great evolutionary divergence may contribute to the potential functional diversity in soybean *SOD* genes. Taken together, we established a foundation for further functional characterization of soybean *SOD* genes in response to alkaline stress in the future.

## Introduction

Abiotic stresses, such as salt, cold and drought, are the main causes that affect plant growth and production. These abiotic stresses disrupt the equilibrium of oxidative reaction, increase the toxic reactive oxygen species (ROS) generation and create oxidative stress (Gill & Tuteja, 2010; Shokri-Gharelo & Noparvar, 2018). To adapt to these toxic ROS, plants have developed a series of enzymatic defense systems, such as catalase (CAT) system, glutathione S-transferase system, glutathione reductase system and superoxide dismutase (SOD) system (Finn et al., 2016). The SOD enzymes, as one of the antioxidant enzymes, play significantly roles in plant against oxidative stress (Geng et al., 2018). The toxic superoxide anion can be reduced by SOD dismutation to molecular oxygen and hydrogen peroxide in plant cells under oxidative stress (Quan et al., 2008).

Previous studies revealed that *SOD* genes were divided into three subfamilies (Kliebenstein, Monde & Last, 1998; Zhou et al., 2017). However, others showed that *SOD* genes could be classified into four subfamilies: MnSOD, FeSOD, Cu/ZnSOD and NiSOD (Gopavajhula et al., 2013). MnSOD, FeSOD and Cu/ZnSOD occur in almost all plants, whereas Ni-containing SOD was found in Streptomyces (Dupont et al., 2008). In *Arabidopsis thaliana*, eight *SOD* genes have been divided into three subfamilies: one MnSOD (*MSD1*), three FeSODs (*FSD1–3*) and Cu/ZnSODs (*CSD1–3*) subfamily, based on their types of prosthetic metals (Kliebenstein, Monde & Last, 1998). Frequently, different *SOD* subfamily genes are distributed to different cellular compartments (Bueno et al., 1995; Corpas et al., 2006). For example, *MnSOD* subfamily genes were mainly observed in mitochondria and also localized in different types of peroxisomes. FeSODs were reported to localize in chloroplasts cytoplasm. Cu/ZnSOD mainly localized in chloroplasts as well as in peroxisomes or cytoplasm. Studies have reported that these subfamily genes play essential roles in response to various environmental stresses. In addition, the plants improve stress tolerance mainly by decreasing the oxidative stress and enhancing the antioxidative defense capacity. For example, overexpression of *OsCu/ZnSOD* gene improved saline-sodic stress resistance in rice by increasing the detoxification capacity of ROS and reducing salt-induced oxidative damage. Ectopic expression of *MnSOD* gene in tomato confers tolerance to salt and oxidative stress (Guan et al., 2017). Furthermore, The *FeSOD* gene from *Arabidopsis* not only increased oxidative stress to transgenic maize, but also played important roles in early chloroplast development (Myouga et al., 2008). However, the previous studies mainly focused on the mechanism of SODs protection in plants against salt, drought and heat stresses, few studies have been reported on the *SOD* gene family in response to alkaline stress, especially in soybean.

Alkaline stress affects plant cells mainly by HCO_3_^−^ , CO_3_^2−^ and high pH, which impose more serious damages than other stresses. Alkaline stress (HCO_3_^−^ and CO_3_^2−^) can inhibit the absorption of NO_3_^−^, Cl^−^ and Fe^2+^, and breaks the ionic balance. The photosynthetic rate, sugar production, N metabolism and amino acid production were also significantly inhibited under alkaline stress (at high pH) (Hu et al., 2015). Further, alkaline stress has affected 434 million ha of land and limited the crop productivity worldwide (Chen et al., 2018), although single form of soybean *SOD* gene has been identified under various stresses (Gopavajhula et al., 2013; Wang et al., 2016). It is necessary to explore the roles of soybean SOD family under environmental stresses, especially under alkaline stress.

In the present study, to comprehensively explore the soybean SOD family, we identified 13 *SOD* genes from *Glycine max* database by using the HMM profile. We further determined their evolutionary relationship, gene structure, conserved domain, chromosomal locations and promoter *cis*-elements. Finally, we identified their expression patterns under alkaline, salt and cold stresses. We further suggested that soybean *SOD* genes possibly participate in responding to alkaline using qRT-PCR analysis.

## Materials and Methods

### Identification of *SOD* family genes in soybean genome

To identify all potential family members of *SOD* in soybean, the known soybean SOD amino acid sequences were used as queries to establish a Hidden Markov model (Gopavajhula et al., 2013), and searched in soybean genome database by using the HMM profile (build 2.3.2) (Finn, Clements & Eddy, 2011). The Pfam and SMART database were used to remove incomplete domains and overlapping genes (Finn et al., 2016). The molecular weight and isoelectric point values of SOD proteins were predicted using online software ExPASy (http://au.expasy.org/tools/pi_tool.html) (Artimo et al., 2012).

### Bioinformatics analysis of *SOD* family genes

The neighbor-joining phylogenetic tree was generated by using software MEGA 5.0 (Kumar et al., 2008). The exon-intron structures were analyzed by the GSDS online server (http://gsds.cbi.pku.edu.cn/) (DuanMu et al., 2015). The conserved motifs were analyzed by MEME online server (http://meme-suite.org/) (Bailey et al., 2009). The multiple sequence alignments were performed using Clustal X program (Larkin et al., 2007). The syntenic analysis was conducted using Circos-0.69 (http://circos.ca/) (Krzywinski et al., 2009). The synonymous (Ks) and nonsynonymous (Ka) substitution rates of syntenic paralogs were calculated using DnaSP software (version 5.10.01) (Krzywinski et al., 2009). The PLANT CARE online software was used to analyses the *cis*-acting elements of promoters (http://bioinformatics.psb.ugent.be/webtools/plantcare/html/) (Lescot et al., 2002).

### Expression patterns of *SOD* family genes based on transcriptome sequencing data

To examine the expression profiles of soybean *SOD* family genes in different tissues, the expression profile data (GSE29163) was obtained from NCBI. The transcriptome data of soybean was downloaded from the NCBI database under cold (GSE117686), salt (GSE57252) and water-deficit (GSE49537) stresses. As there is limited information exist on soybean in response to alkaline stress, we downloaded wild soybean transcriptome data under alkaline stress. The hierarchical clustering trees of *SOD* family genes were generated using TM4: MeV4.9 software (Saeed et al., 2006).

### Plant material, growth condition and alkaline stress treatment

The soybean (DN50) seeds were treated with 75% ethanol for 1 min and washed with sterile water before germination 2 days. Then, seedlings were grown in Hoagland nutrient solutions at 70–80% relative humidity, 22–28 °C room temperature and 8 h dark/16 h light. Twelve days after sowing, soybean seedlings were transferred into Hoagland solutions with 50 mm NaHCO_3_ for 0, 6 and 12 h (DuanMu et al., 2015). The roots and leaves were harvested as three biological replicates.

### Transcript data analysis of *SOD* family genes under alkaline stress

Total RNA was extracted from soybean using the plant total RNA isolation kit (TIANGEN, Beijing, China), and the cDNAs were synthesized using the TransScript All-in-One First-Strand cDNA synthesis SuperMix for qPCR kit (TransGen Biotech, Beijing, China). qRT-PCR assays were performed using UtraSYBR Mixture (low ROX) and ABI 7500 sequencer. The *GmGADPH* was used as internal control (Huis, Hawkins & Neutelings, 2010). The *SOD* genes and *GmGADPH* primers are listed in supplementary Table S1. The three biological replicates were obtained and expression levels were calculated using 2^−ΔΔCt^ method and Student’s *t*-test (Livak & Schmittgen, 2001).

## Results

### Identification and phylogenetic analysis of *SOD* genes in soybean

**Figure 1:**
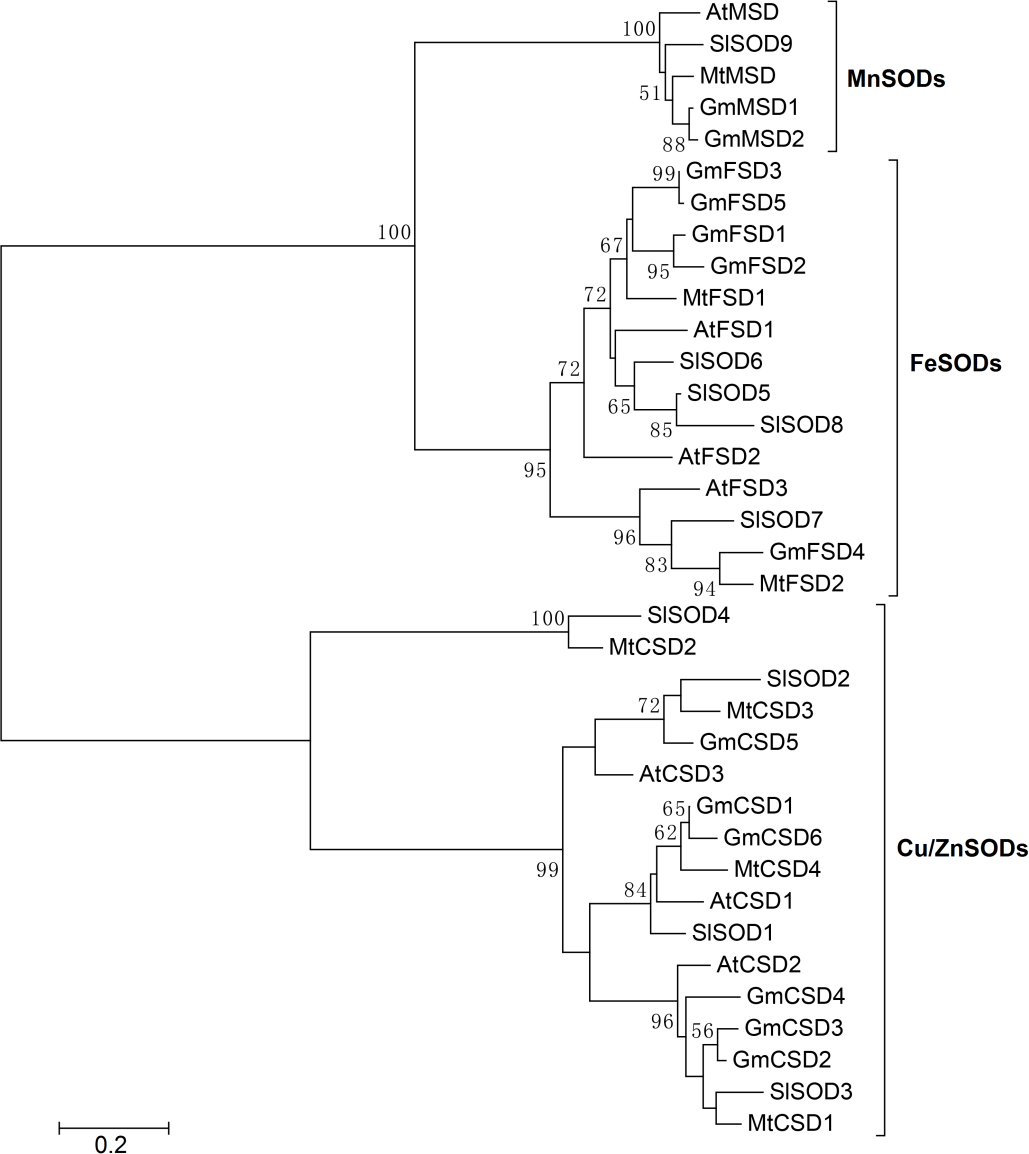
Phylogenetic analysis of *SOD* genes in soybean, *Arabidopsis*, *Medicago truncatula* and *Solanum lycopersicum*. The phylogenetic tree was constructed by the neighbor-joining method using MEGA 5.0. The bootstrap values were 1,000 replications for major branches. SOD family genes have been divided into three subfamilies.

In soybean, ten SOD isoenzymes were identified in different tissues (Wang et al., 2016). For example, one MnSOD isoenzyme was mainly detected in stems and seeds. Four Cu/ZnSOD isoenzymes were detected in roots, leaves, stems and seeds. While, only five FeSOD isoenzymes was mainly detected in leaves. To further explore the *SOD* genes in *G. max*, we used SOD amino acid as query sequences to search the *G. max* database from NCBI. A total of 19 SOD candidate proteins were obtained. All candidate proteins were subjected to Pfam and SMART database to remove incomplete domains and overlapping genes. As a result, we obtained 13 non-redundant *SOD* genes in soybean. To confirm the classification and evolutionary relationships of soybean SOD family members, the full-length protein sequences of GmSODs were used to construct a neighbor-joining phylogenetic tree with *Arabidopsis*, *Medicago truncatula* and *Solanum lycopersicum* SOD family members. The results revealed that the soybean SOD family members were classified into three subfamilies, including MnSODs (*GmMSD1–2*), FeSODs (*GmFSD1–5*) and Cu/ZnSODs (*GmCSD1–6*), which is consistent with previous studies (Fig. 1). In addition, compared with previous study (Wang et al., 2016), we identified three more SOD genes in soybean.

As shown in Table 1, these GmSOD family members were chosen for further protein information analyses, including the exon numbers, length of CDS, protein sequence lengths, molecular weights (MW), and theoretical isoelectric points (*p*I) values. The protein sequence length ranged from 152 (GmCSD1 and GmCSD6) to 314 (GmFSD1) amino acids (aa). The MW varied from 15.19 (GmCSD6) to 35.86 (GmFSD1) kDa and the *p*I values ranged from 5.22 (GmMSD1) to 8.57 (GmFSD2). The Cu/ZnSODs subfamily members had a lower protein sequence length and MW than others. The MnSODs subfamily members appeared with higher *p*I values. This finding indicated that three subfamilies may displayed a great diversity in soybean.

**Table 1:** Protein information of *SOD* genes in soybean.

Gene ID	Gene name	Exon number	Length of CDS (bp)	Amino acid residues	MW (kDa)	*p*I	Chromosome	Domain
Glyma.04G221300	*GmMSD1*	6	723	240	26.54	8.57	4	alpha-hairpin domain, C-terminal domain
Glyma.06G144500	*GmMSD2*	6	726	241	26.70	8.56	6	alpha-hairpin domain, C-terminal domain
Glyma.02G087700	*GmFSD1*	9	945	314	35.86	5.59	2	alpha-hairpin domain, C-terminal domain
Glyma.10G117100	*GmFSD2*	9	933	310	35.26	5.22	10	alpha-hairpin domain, C-terminal domain
Glyma.10G193500	*GmFSD3*	9	735	244	27.51	5.45	10	alpha-hairpin domain, C-terminal domain
Glyma.20G050800	*GmFSD4*	9	918	305	35.10	6.40	20	alpha-hairpin domain, C-terminal domain
Glyma.20G196900	*GmFSD5*	9	747	248	27.84	5.60	20	alpha-hairpin domain, C-terminal domain
Glyma.03G242900	*GmCSD1*	7	459	152	15.23	5.59	3	Copper/zinc superoxide dismutase (SODC)
Glyma.11G192700	*GmCSD2*	8	630	209	21.64	5.87	11	Copper/zinc superoxide dismutase (SODC)
Glyma.12G081300	*GmCSD3*	8	615	204	20.80	5.79	12	Copper/zinc superoxide dismutase (SODC)
Glyma.12G178800	*GmCSD4*	8	552	183	18.62	6.28	12	Copper/zinc superoxide dismutase (SODC)
Glyma.16G153900	*GmCSD5*	7	504	167	17.17	7.19	16	Copper/zinc superoxide dismutase (SODC)
Glyma.19G240400	*GmCSD6*	7	459	152	15.19	5.27	19	Copper/zinc superoxide dismutase (SODC)

### Phylogenetic and gene structure analysis of *SOD* genes

**Figure 2:**
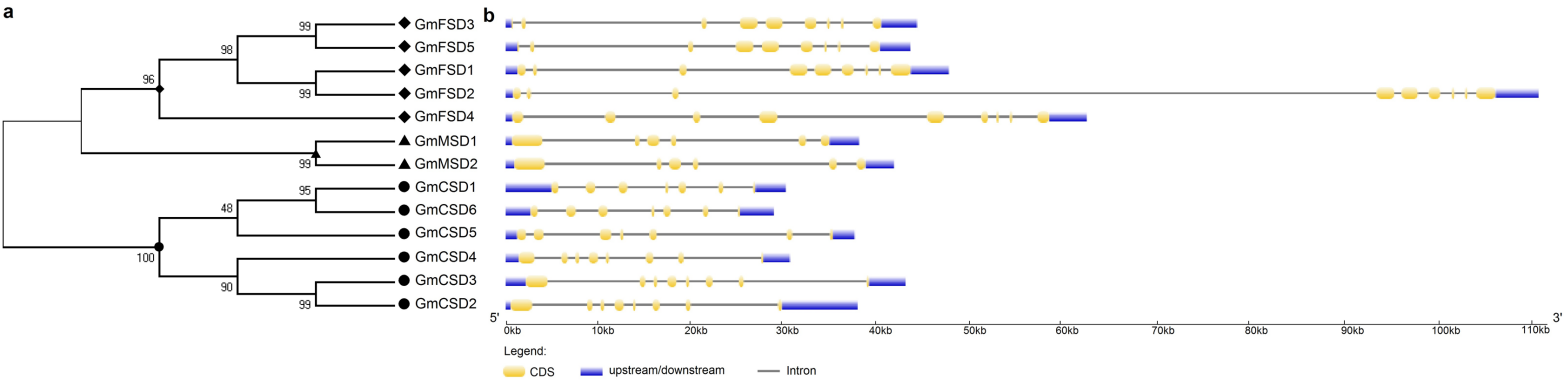
Phylogenetic and exon-intron structure analyses of *SOD* genes. (A) The phylogenetic tree was produced by the neighbor-joining method using MEGA 5.0. The bootstrap values were 1,000 replications for major branches. (B) Exon-intron structure analysis of soybean *SOD* genes by using GSDS online tools. The CDSs, untranslated regions and introns are described by yellow boxes, light blue boxes and black lines, respectively.

To further investigate the evolutionary relationship of *SOD* genes in soybean, we constructed a neighbor-joining phylogenetic rootless tree with the full-length protein sequences of GmSODs. The results suggested that three SOD subfamilies have a distant evolutionary relationship (Fig. 2A). However, we found that each SOD subfamilies have high bootstrap support pairs, such as *GmFSD3* and *GmFSD5*, *GmMSD1* and *GmMSD2*, *GmCSD2* and *GmCSD3*. This indicated that each soybean SOD subfamilies was evolutionarily conserved, even though three subfamilies displayed a distant evolutionary relationship.

The diversity of gene structure is mainly influenced by the evolution of multigene families (Mercereau-Puijalon, Barale & Bischoff, 2002; Pellicer et al., 2018). To further explore the structural diversity of *SOD* genes, the characteristics of exon-intron structures were analyzed by the GSDS online server. As shown in Fig. 2B, different subfamilies displayed variation in exon-intron structures. For example, the MnSODs subfamily comprised of six exons as well as exhibited a similarity in genomic structure. The FeSODs subfamily members consisted of nine exons. We also noticed that *GmFSD2* exhibited a longer genomic structure than 10 KB. However, three Cu/ZnSODs subfamily members (*GmCSD1*, *GmCSD5* and *GmCSD6*) possessed seven exons, while others appeared with eight exons. These results further confirmed that the different SOD subfamilies diverged greatly in soybean.

### Conserved domain analysis of SOD proteins

**Figure 3:**
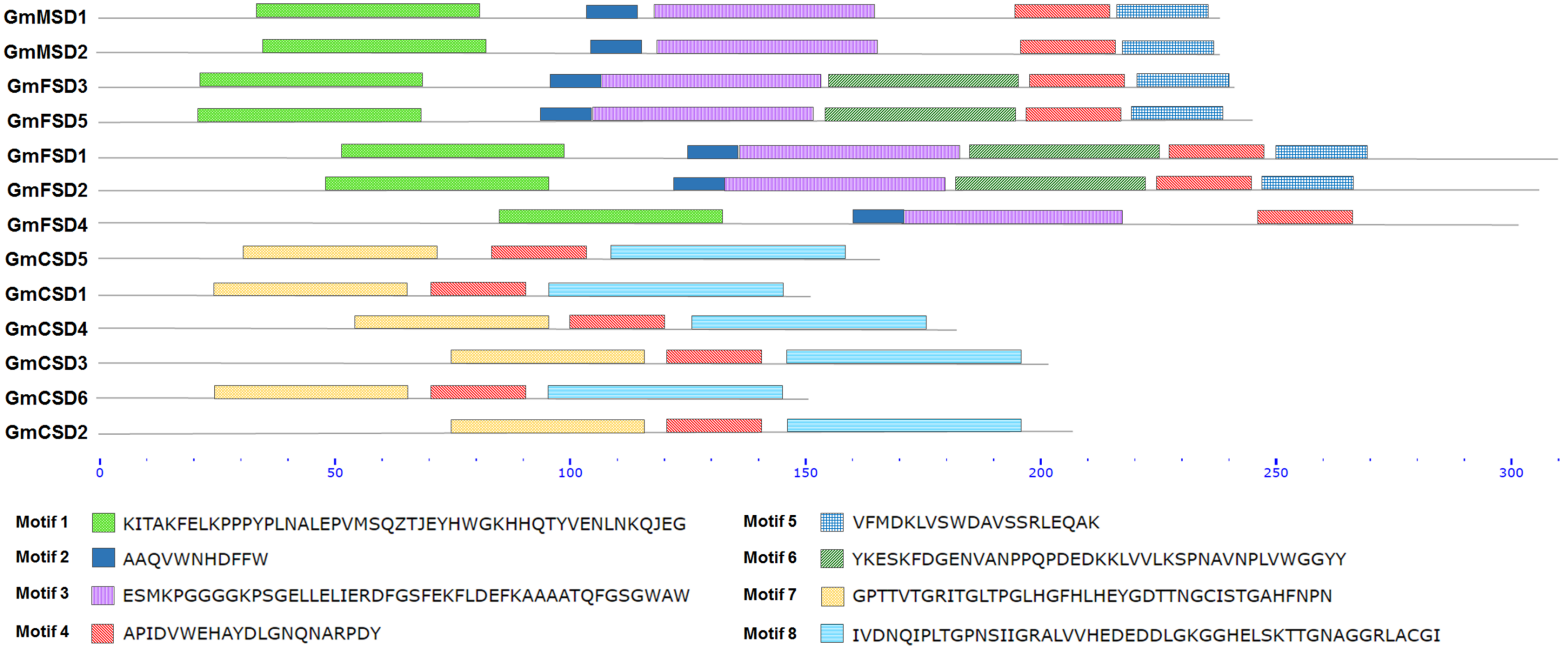
Conserved domain analysis of SOD family proteins. The conserved motifs were predicted using the MEME online server. The different conserved motifs were marked by different colors. The protein sequences of eight different motifs commonly observed by MEME online server.

For identification of the conserved domains of SOD proteins in soybean, the conserved motifs were analyzed by the MEME online server. Our results showed that eight predicted conserved motifs were identified (Fig. 3). Among them, all proteins in Cu/ZnSODs subfamilies contained motifs 4, 7 and 8. Motifs 1, 2, 3, 4 and 5 were detected in MnSODs subfamily members. Motifs 1, 2, 3, 4, 5 and 6 were discovered in all FeSODs subfamily members, except *GmFSD2*. It is noteworthy that motif 1 was detected in the N-terminal domain (alpha-hairpin domain) of MnSODs and FeSODs subfamily members. Besides, motifs 2, 3, 4 and 5 were also detected in the C-terminal domain. The motifs 4, 7 and 8 were also detected in the copper/zinc SOD domain of Cu/ZnSODs subfamily members (Figs. S1 and S2). These results indicated that the MnSODs and FeSODs subfamily members showed high similarities in conserved sequences. However, the Cu/ZnSODs subfamilies displayed wide divergence with other two subfamilies, which further verify the potential diversity of functional divergence in soybean *SOD* genes from different subfamilies.

### Chromosomal locations and syntenic analysis

**Figure 4:**
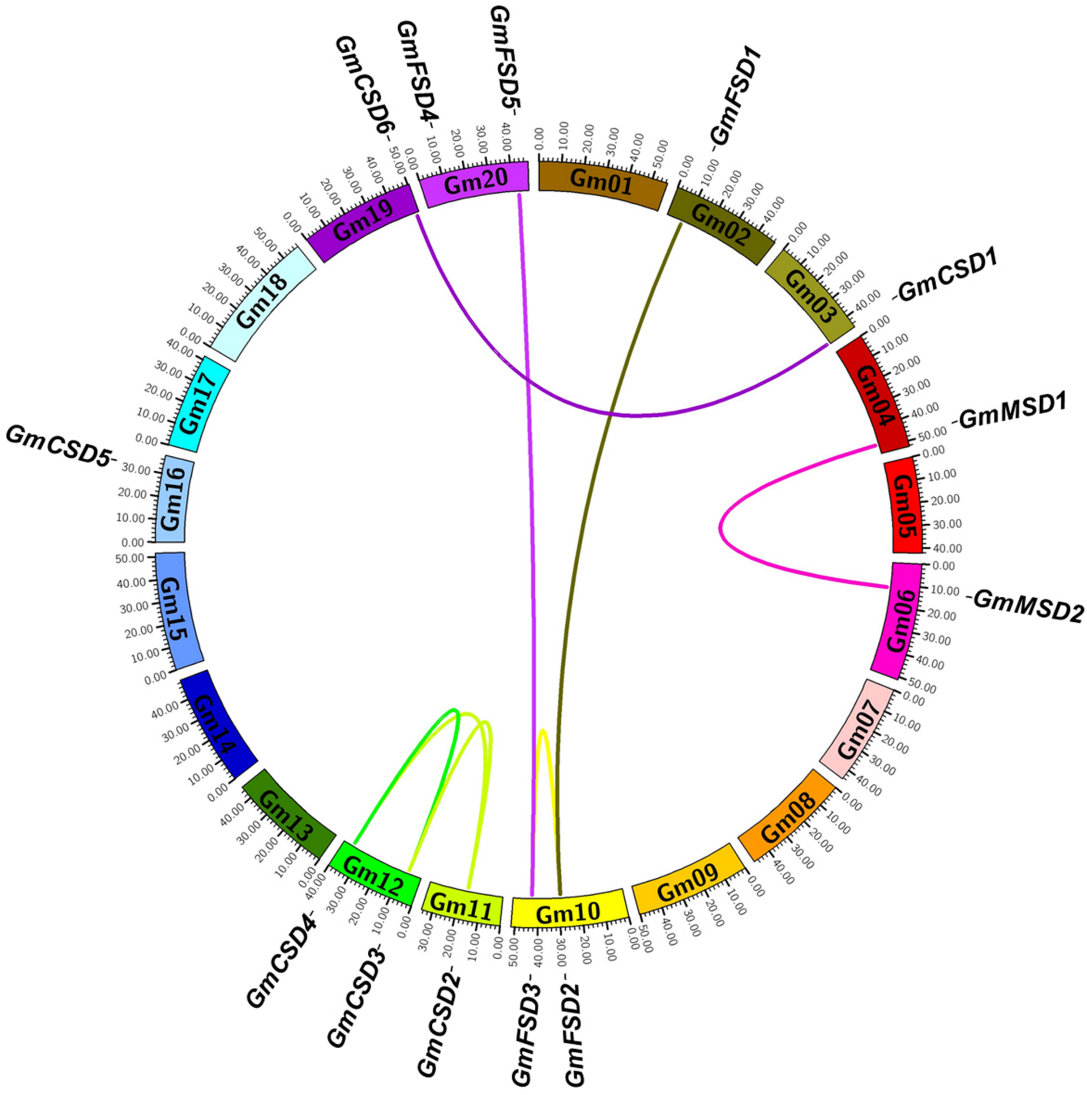
Syntenic analysis of *SOD* family genes in soybean. The chromosomes are indicated as a circle. The duplication pairs are connected by lines.

To determine the genomic distribution of *SOD* genes, we identified their gene locations on the chromosomes and potential genome duplication events using the syntenic analysis. The thirteen *SOD* genes were randomly distributed among ten chromosomes, and each chromosome had one or two genes (Fig. 4). Gene duplication plays important role in plant functional diversity and evolutionary mechanism (Bowers et al., 2003). To detect the potential genome duplication events, a total of eight pairs of SOD syntenic paralogs were found in soybean genome, except *GmCSD5*. The results indicated that the soybean SOD family existed a high gene family expansion. Meanwhile, we found that the duplication pairs also exhibited a high evolutionary relationship in phylogenetic analysis (Fig. 2A).

The non-synonymous/synonymous substitution (Ka/Ks) rates were used to detect the positive or negative selection of gene family expansion (Kaehler, Yap & Huttley, 2017) . To further investigate whether selective pressure was associated with the *SOD* family genes, the Ka/Ks rates were calculated for the full-length CDS sequences of eight duplication pairs (Table S2). Previous studies reported that Ka/Ks < 1 indicates a negative selection and Ka/Ks > 1 indicates a positive selection (Wang et al., 2005). The results showed that the Ka/Ks rates for six SOD duplication pairs were more than 1. However, two SOD duplication pairs were less than 1. This finding indicated that the soybean *SOD* genes had experienced positive and negative selection pressure after the duplication events.

### Analysis of *cis*-acting elements of *SOD* gene promoters

**Figure 5:**
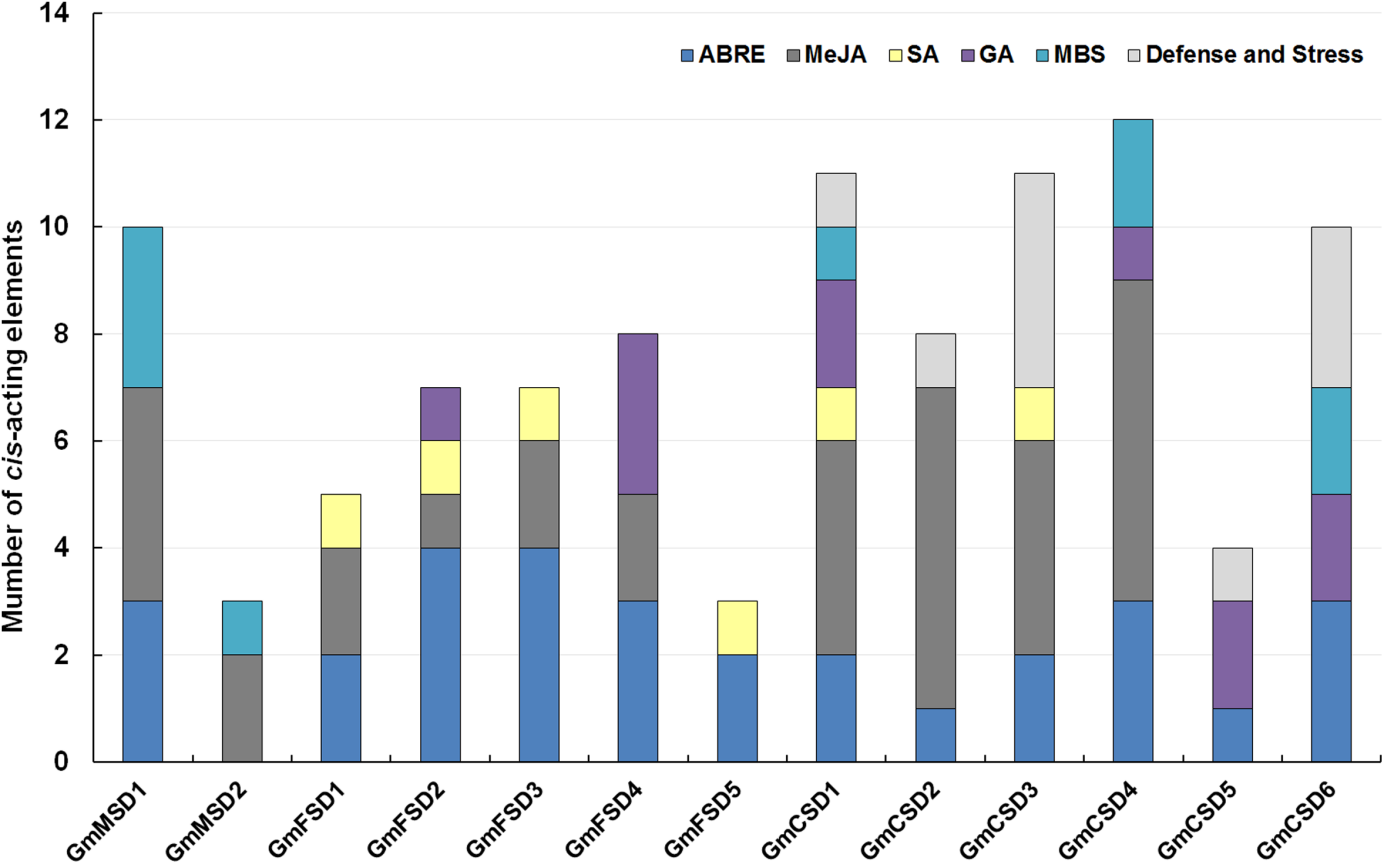
Analysis of *cis*-acting elements of putative SOD promoters related to stress responses. The different *cis*-elements are present with different colors.

The *cis*-acting elements play significant roles in determine the regulatory roles under various stresses (Kimotho, Baillo & Zhang, 2019). In this study, to explore the potential roles of the soybean *SOD* genes under environmental stresses, we analyzed the *SOD* gene promoters coupled with 3 KB genomic sequence upstream regions of the ATG using PlantCARE online tool (Sun et al., 2014). The *SOD* family genes possessed a variety of putative abiotic stress and hormone-related responsive elements, including ABA (ABRE), Methyl jasmonate (MeJA), Salicylic acid, Gibberellin, MBS (Drought) and Defense and Stress responsive elements (Fig. 5; Table S3). Among them, almost all *SOD* family genes contained ABA-responsive elements, except *GmMSD2*. Ten of *SOD* family genes had MeJA-responsive elements. However, the *GmMSD2* and *GmFSD5* only contained two responsive elements, respectively. We also found that only *Cu/ZnSODs* subfamily genes contained Defense and Stress responsive elements. Collectively, the results suggested that *SOD* family genes might be related to the responses of abiotic stresses and hormone stimuli. Also, these genes might have potential functional diversity because they contained different responsive elements.

### Expression profiles of *SOD* genes in soybean tissues

**Figure 6:**
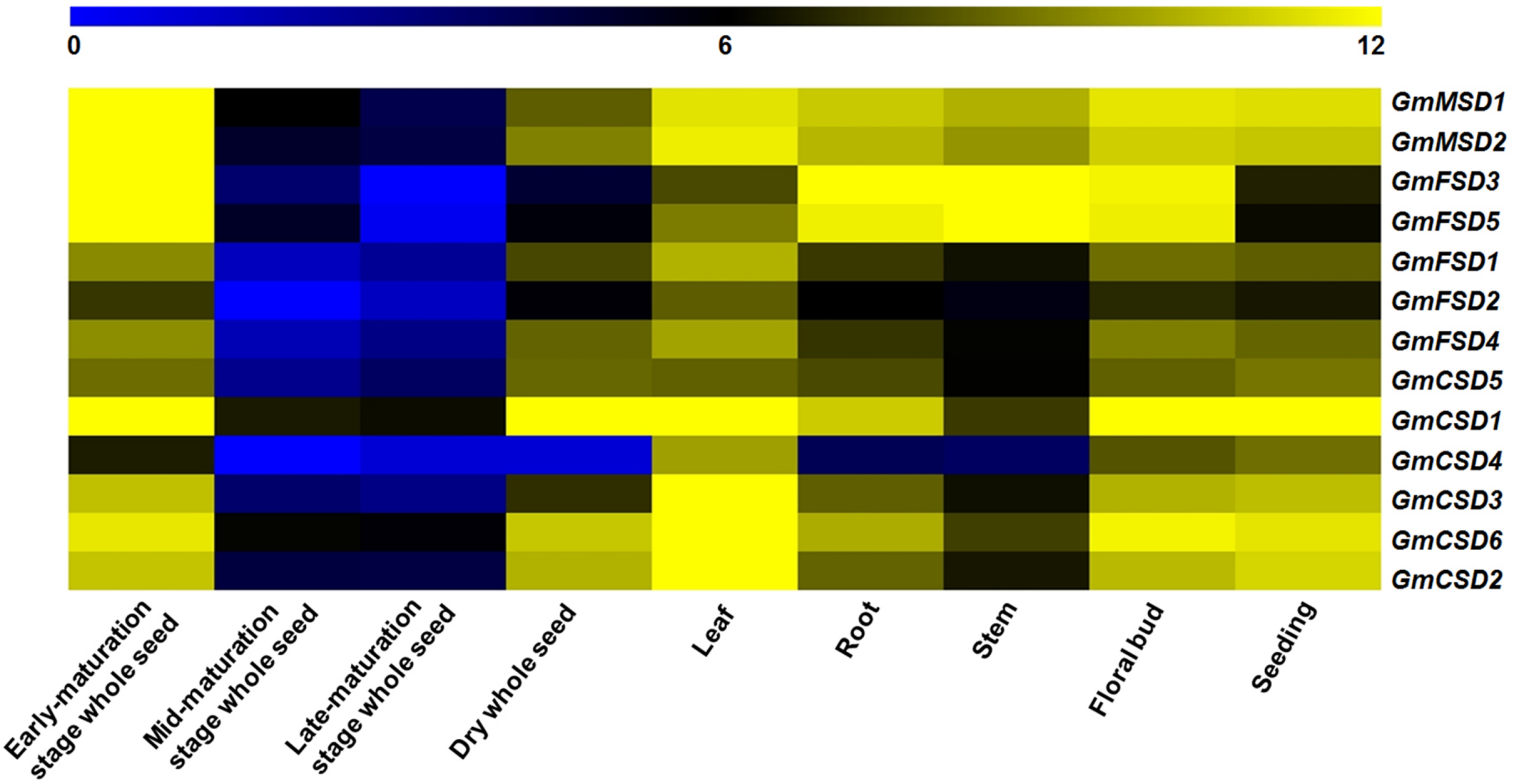
Expression patterns of *SOD* family genes in different soybean tissues. The RNA-seq data (GSE29163) were downloaded from the NCBI. All expression data were analyzed by Log_2_ scaled. The heat map was generated using TM4: MeV4.9 software. The color scale represents the expression values: blue indicates low levels and yellow represents high levels.

To investigate the potential function of *SOD* genes in soybean growth and development, we analyzed their expression in nine tissues using RNA-seq data (Chen et al., 2012). The results showed that the numbers of expressed soybean *SOD* genes in different tissues exhibited higher variations (Fig. 6). For example, *GmMSD1*, *GmMSD2*, *GmFSD3*, *GmFSD5* and *GmCSD1* showed higher expressions in early-maturation stage whole seed. *GmCSD1*, *GmCSD2*, *GmCSD*3 and *GmCSD6* had higher expression levels in leaf. In addition, the *GmCSD1* displayed the higher expression level in five tissues, which is consistent with the cucumber *CSD1* gene studies (Zhou et al., 2017). The *GmFSD2* showed an extremely lower expression levels than others in nine tissues. However, almost all the soybean *SOD* genes had the lowest expression level in mid- and late-maturation stage whole seed. In conclusion, these results indicated that soybean *SOD* genes showed special tissue expression profiles, indicating their potential divergent functions in soybean growth and development.

### Expression analysis of soybean *SOD* genes in response to abiotic stresses

**Figure 7:**
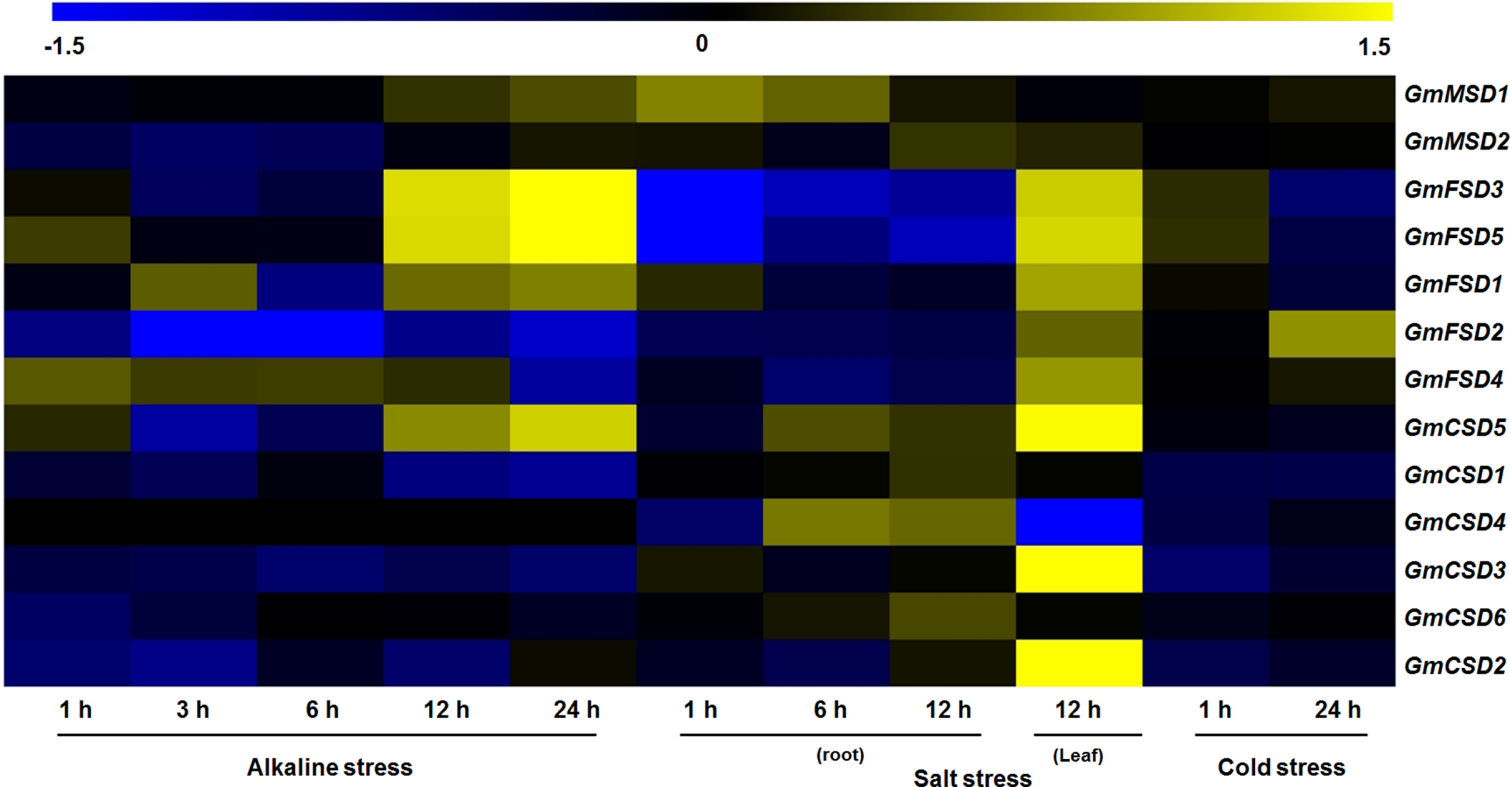
Expression analysis of soybean *SOD* genes in response to various abiotic stresses. The wild soybean transcriptome sequencing data were used to investigate the expression pattern of soybean *SOD* genes under alkaline stress. Expression of soybean *SOD* genes were downloaded from the soybean transcriptome sequencing data under salt (GSE57252) and cold (GSE57252) stresses. The heat map was generated using TM4: MeV4.9 software. The color scale represents the expression values: blue indicates low levels and yellow represents high levels (|Log_2_ fold change| > 1, *P* < 0.05).

Previous studies have showed that the *SOD* genes play significant roles in plant responses to various stresses (Jiang et al., 2019). To investigate the potential roles of soybean *SOD* genes in various abiotic stress responses, we identified their expression patterns under alkaline, salt and cold stresses using transcriptome sequencing data. However, there is limited information exist on soybean in response to alkaline stress, we downloaded wild soybean transcriptome data under alkaline stress. As shown in Fig. 7, the heat map revealed that four and six of soybean *SOD* genes were differentially expressed (|Log_2_ fold change| > 1, *P* < 0.05) under alkaline and salt stresses, respectively. However, only *GmFSD2* displayed slightly up-regulation under cold stress. Among them, we found that *GmFSD3*, *GmFSD5* and *GmCSD5* were all up-regulated under alkaline and salt stresses, indicating that they might act as positive regulators. It is worth noting that *GmFSD3* and *GmFSD5* showed differential expression in soybean leaves and roots under salt stress. This finding implied that these two genes may be involved in different signaling pathways in leaves and roots under salt stress.

### Expression analysis of *SOD* genes in response to alkaline treatment

**Figure 8:**
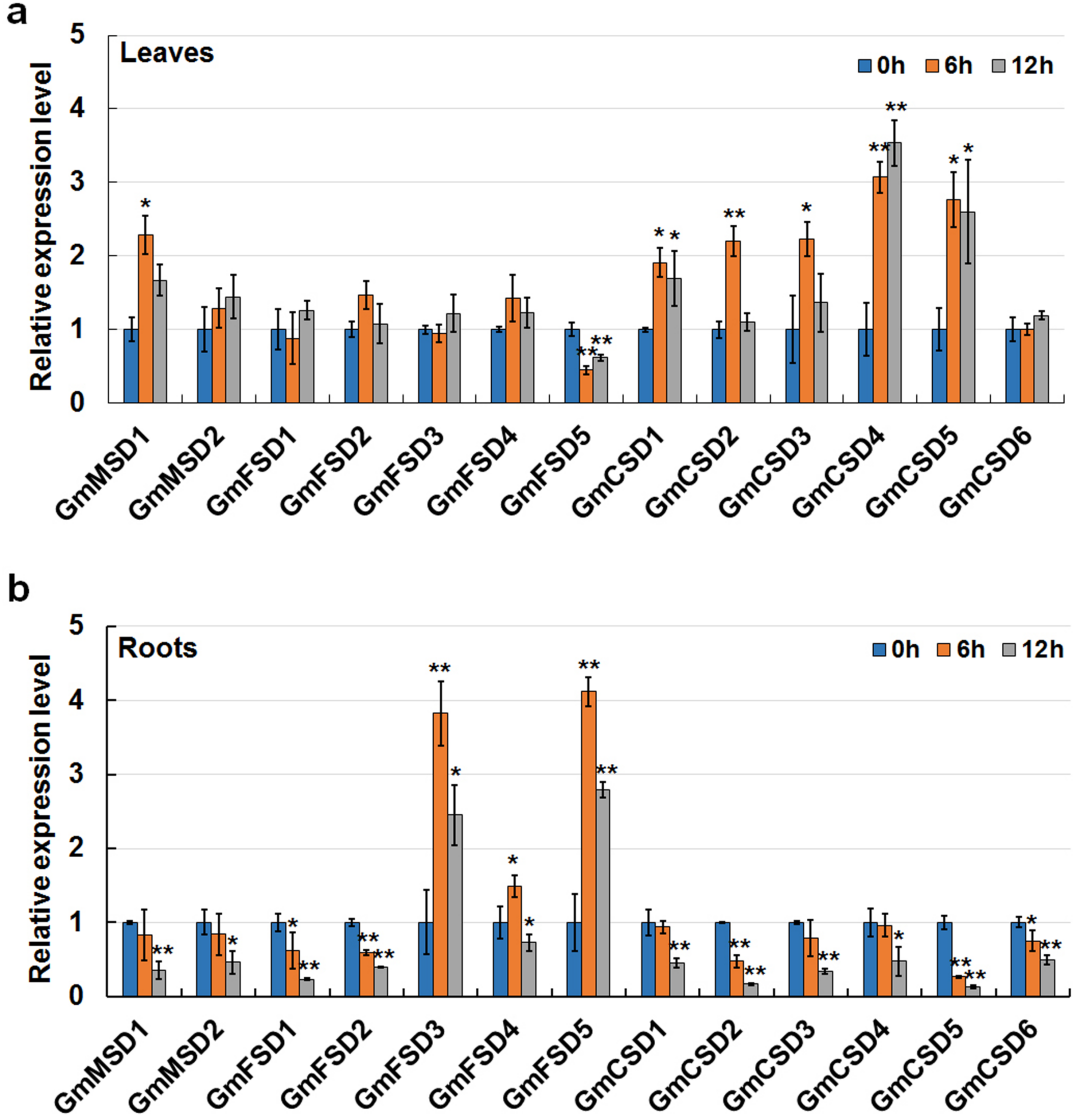
Expression analysis of soybean *SOD* genes in response to alkaline stress in leaves (A) and roots (B) using qRT-PCR assays. Twelve days soybean seedlings were treated with 50 mm NaHCO_3_ for 0, 6 and 12 h. The mean values were from three independent biological replicates. Statistical analyses were performed by Student’s *t*-test (*P < 0.05 and **P < 0.01).

Saline-alkali soil is considered as a major threat to crop growth and yields. However, only a few studies have focused on the mechanisms of plants respond to alkaline stress. In this study, to further confirm the soybean *SOD* genes in response to alkaline treatment, we detected their expression patterns in roots and leaves under 50 mm NaHCO_3_ treatment by using qRT-PCR. As shown in Fig. 8A total of six genes were up-regulated expressed and only *GmFSD5* gene was down-regulated in soybean leaves under alkaline treatment. Three genes (*GmFSD3*, *GmFSD4* and G*mFSD5*) were significantly induced in soybean roots under alkaline treatment, whereas others showed down-regulated expression, especially at 12 h. Among them, *GmFSD5* had contrary expression pattern in leaves and roots, indicating this gene may be involved in different pathways.

In addition, we found that *FeSODs* and *Cu/ZnSODs* subfamily genes displayed different roles in roots and leaves after alkaline treatment. For example, only three *FeSODs* subfamily genes (*GmFSD3*, *GmFSD4* and G*mFSD5*) were significantly induced (*P* < 0.05 or *P* < 0.01) in roots. Five *Cu/ZnSODs* subfamily genes (*GmCSD1/2/3/4/5*) were up-regulated in leaves. However, almost all *FeSODs* subfamily genes didn’t express significantly in leaves, except *GmFSD5* which was down-regulated expressed. To conclude, qRT-PCR confirmed the results that soybean *SOD* genes possibly participate in responses to alkaline.

## Discussion

Antioxidant enzymes SOD play significantly roles in plant response to abiotic stresses by reducing the molecular oxygen and hydrogen peroxide in plant cells. The plant *SOD* family genes have been identified and characterized in some species at the genome-wide level (Feng et al., 2016; Verma, Lakhanpal & Singh, 2019). However, the roles of soybean *SOD* family genes in regulating various abiotic stresses, especially alkaline stress, are rarely reported. Hence, our research conducted a comprehensive exploration of soybean SOD family, and mainly identified potential roles of soybean *SOD* genes in response to alkaline stress.

The *SOD* family genes were reported to be divided into three subfamilies, and each subfamily genes frequently located in different cellular compartments (Feng et al., 2016). This indicated that *SOD* family genes may display evolutionarily divergence. Here, we found several evidences to confirm the divergent evolution in soybean SOD family. Firstly, according to the results of the phylogenetic tree, the soybean *SOD* family genes were divided into three subfamilies (Fig. 1). In addition, each subfamily varied markedly in protein sequence length and theoretical *p*I values (Table 1). Secondly, the different subfamilies displayed different exon-intron structures and exon numbers (Fig. 2B). For example, *MnSODs* and *FeSODs* subfamily genes contained six and nine exons, respectively. However, *Cu/ZnSODs* subfamily genes possessed seven to eight exons. These results are in line with previous reports (Song et al., 2018), and further confirmed that three subfamilies may displayed a great diversity in soybean. Thirdly, the conserved motifs further revealed the varied widely from different subfamilies, especially MnSODs and Cu/ZnSODs subfamily (Fig. 3), which consistent with previous study (Wang et al., 2017). In conclusion, the above results confirmed SOD subfamilies displayed evolutionary divergence in soybean. Consistently, previous study showed that Cu/Zn-SODs evolved independently in *Gossypium hirsutum* (Wang et al., 2017). And MnSODs and FeSODs have evolved with common ancestral enzymes. This evidence could explain the similar motif patterns were found in MnSODs and FeSODs subfamilies (Fig. 3).

On the other hand, the great evolutionary divergence may contribute to the potential functional diversity in soybean SOD family. Among them, only *Cu/ZnSODs* subfamily genes contained Defense and Stress responsive elements. The *GmMSD2* and *GmFSD5* exhibited completely different abiotic stresses and hormone-related responsive elements (Fig. 5; Table S3). Moreover, the numbers of expressed soybean *SOD* genes in different tissues showed a lot of variations (Fig. 6). For example, four Cu/ZnSOD genes (*GmCSD1*, *GmCSD2*, *GmCSD3* and *GmCSD6*) had higher expression levels in leaf. *GmCSD1* displayed the higher expression levels in five tissues, while *GmFSD2* showed opposite expression levels. In line with this, *VvCSD2*, *VvCSD4* and *VvCSD5* exhibited different expression patterns as compared with *VvFSD1* and *VvFSD2* in all the tested tissues in Grapevine (Hu et al., 2019). Also, they displayed different tissue expression profiles and *cis*-acting elements, indicating their potential divergent functions in soybean growth and responses to environmental stresses.

Even though the soybean SOD family displayed a great evolutionary divergence, however the duplication events also were detected in each SOD subfamilies. A total of eight pair of duplication genes were found in soybean genome by using syntenic analysis (Fig. 4). The duplication pairs also existed a high evolutionary relationship in phylogenetic analysis (Fig. 2A). In plants, the duplication events help plants to adapt to diverse environments via gene generation, loss and rearrangement (Faillace et al., 2019; Shang et al., 2013). Thus, our results indicated that soybean *SOD* family genes possess gene duplication to adapt to environmental conditions. And *SOD* genes have experienced positive and negative selection pressure after the duplication events according to the Ka/Ks rates (Table S2).

Three subfamilies of SODs have been reported to eliminate toxic ROS caused by abiotic stresses (Wu et al., 1999). To further explore the roles of *SOD* genes in soybean in relation with environment stress responses, we examined the expression patterns using transcriptome sequencing data, including salt, cold and alkaline stresses (Fig. 7). The results revealed that four and six of soybean *SOD* genes were significantly induced under alkaline and salt stresses, respectively. Similarly, studies have reported that *SOD* genes played positive roles in other plants (Feng et al., 2016). Whereas, almost all the expression levels of soybean *SOD* genes displayed no significant change under cold stress, except for a slightly up-regulation (|Log_2_ fold change| > 1) of *GmFSD2*. This indicated that the soybean *SOD* genes may play different roles under cold stress, which also consistent with previous research (Song et al., 2018). In addition, we also examined the expression patterns of soybean *SOD* genes under water-deficit stress (Fig. S3). The results showed that few soybean *SOD* genes showed significant up-regulated expressions, while five genes were down-regulated. In conclusion, these results suggested that soybean *SOD* genes may play different roles under various environment stresses.

Alkaline stress can impact crop growth and yields via disturbance in ionic balance and inhibition in organic synthesis. In this study, we focus on the potential roles of soybean *SOD* genes in response to alkaline treatment in roots and leaves by using qRT-PCR (Fig. 8). Firstly, qRT-PCR confirmed the results that soybean *SOD* genes may play positive roles in response to alkaline stress. For example, six and three soybean *SOD* genes were significantly induced (*P* < 0.05 or *P* < 0.01) in leaves and roots under alkaline treatment, respectively. Secondly, we found that different subfamilies might display different roles in leaves and roots under alkaline stress. For example, *FeSODs* and *Cu/ZnSODs* subfamily genes showed opposite expression patterns. Three *FeSOD* genes (*GmFSD3*, *GmFSD4* and G*mFSD5*) were significantly induced in roots but not in leaves. In line with this, five *Cu/ZnSODs* subfamily genes (*GmCSD1/2/3/4/5*) were only up-regulated in leaves. These results also consistent with the expression patterns in leaves and roots under salt stress (Fig. 7). In addition, these results also confirmed the hypothesis that the great evolutionary divergence may contribute to the potential functional diversity in soybean *SOD* genes.

## Conclusions

In conclusion, we identified 13 potential soybean *SOD* genes, which could be classified into three subfamilies: MnSODs (*GmMSD1–2*), FeSODs (*GmFSD1–5*) and Cu/ZnSODs (*GmCSD1–6*). We confirmed that SOD subfamilies displayed a great evolutionary divergence in soybean by gene structure, conserved domains and phylogenetic analysis. Furthermore, we identified that *SOD* genes showed special tissue expression profiles, indicating their potential divergent functions in soybean growth and development. *Cis*-acting elements analysis and expression profiles under environmental stresses suggested that *SOD* family genes might be related to the responses of abiotic stresses and hormone stimuli. Moreover, we focused on the *SOD* genes in response to alkaline stress by qRT-PCR, and found that different *SOD* subfamily genes could be involved in different roles in response to alkaline stress. Taken together, we established a foundation for further functional characterization of soybean *SOD* genes in response to alkaline stress in the future.

## Supplemental Information

10.7717/peerj.8457/supp-1 Supplemental Information 1 Raw data: The amino acid sequences of SOD in soybean, *Arabidopsis*, *Medicago truncatula* and *Solanum lycopersicum*.

10.7717/peerj.8457/supp-2 Supplemental Information 2 Raw data: *cis*-acting elements of putative SOD promoters.

10.7717/peerj.8457/supp-3 Supplemental Information 3 Raw data: Expression patterns of SOD family genes based on the transcriptome sequencing data in different soybean tissues.

10.7717/peerj.8457/supp-4 Supplemental Information 4 Raw data: Expression analysis of soybean SOD genes in response to various abiotic stresses based on the transcriptome sequencing data.

10.7717/peerj.8457/supp-5 Supplemental Information 5 Raw data: Expression analysis of SOD genes in response to alkaline treatment.

10.7717/peerj.8457/supp-6 Supplemental Information 6 Raw data: The sequences of genes for non-synonymous and synonymous substitution (KaKs) rates analysis.

10.7717/peerj.8457/supp-7 Supplemental Information 7 Raw data: Expression patterns of the SOD families in soybean based on the transcriptome sequencing data under water-deficit stress.

10.7717/peerj.8457/supp-8 Supplemental Information 8 Gene-specific primers used in this study.

10.7717/peerj.8457/supp-9 Supplemental Information 9 Ka/Ks and divergence analysis of SOD paralogous in soybean.

10.7717/peerj.8457/supp-10 Supplemental Information 10 The information of putative *cis*-acting elements in *SOD* promoters from soybean.

10.7717/peerj.8457/supp-11 Supplemental Information 11 Comparison of the amino acid sequences of SOD families in soybean. The copper/zinc SOD domain of Cu/ZnSODs subfamily members are marked with blue box. The alpha-hairpin domain and C-terminal domain of MnSODs and FeSODs subfamily members are marked with red and black boxes, respectively.

10.7717/peerj.8457/supp-12 Supplemental Information 12 Conserved domain analysis of SOD families in soybean. The motifs of SOD families in soybean are marked with black box.

10.7717/peerj.8457/supp-13 Supplemental Information 13 Expression patterns of the SOD families in soybean based on the transcriptome sequencing data under water-deficit stress. The color scale represents the expression values: blue indicates low levels and yellow represents high levels.

## Additional Information and Declarations

### Data Availability

The following information was supplied regarding data availability: The raw data is available in the Supplemental Files.
