# Supplementary material for: Genome-wide identification and characterization of the soybean SOD family during alkaline stress
Source: PeerJ. 2020 Feb 5;8:e8457. doi: 10.7717/peerj.8457 (PMC7007734; doi:10.7717/peerj.8457)
Supplement: Supplemental Information 8 [file peerj-08-8457-s008.docx]

**Table S1. Gene-specific primers used in this study**

| Gene name | Primer sequence (5’-3’) |  |
| --- | --- | --- |
| *GmMSD1* | Forward: CCATTGACACACATTTTGGCT | |
|  | Reverse: AACATTCTTGTACTGTAAGTAGTACGCA | |
| *GmMSD2* | Forward: TAACAGCAGCGGCTACTCATT | |
|  | Reverse: TTCACCACCTCCTTCACGAA | |
| *GmFSD1* | Forward: TGTTATCGCAATCCACTGCC | |
|  | Reverse: GTCCTATGATGCTTCCCCCA | |
| *GmFSD2* | Forward: CCTTCCAGTGCCGCTTCTAC | |
|  | Reverse: AGTCCTGTGATGCTTCCCCC | |
| *GmFSD3* | Forward: GGGGAAACACCACAAGACATAT | |
|  | Reverse: AAGCCCATCCTGAACCAAAT | |
| *GmFSD4* | Forward: GAGAGAAGAGAAACGACTGGAGAT | |
|  | Reverse: CAAGACACAAGGTGGTTCATAAAT | |
| *GmFSD5* | Forward: GGGTGGGCTTGGCTAGCATA | |
|  | Reverse: AAGGGGGTTCACAGCATTGG | |
| *GmCSD1* | Forward: AGCCTGGTCTCCATGGTTTC | |
|  | Reverse: CGCCCTTCCTATGATGTTGTTT | |
| *GmCSD2* | Forward: TCCCCAATCCATAACGCTTT | |
|  | Reverse: CACCCATTTGTCGTATCACCA | |
| *GmCSD3* | Forward: GTTCCGACCTCATCCCCTTC | |
|  | Reverse: GCTCCTGTTGATATACACCCATTT | |
| *GmCSD4* | Forward: TCCCGCCACTACCAAGAAAG | |
|  | Reverse: GGAGTAAGACCAGAAACACGAACA | |
| *GmCSD5* | Forward: CGGAGACAACAACATTAGAGGC | |
|  | Reverse: GAACGGATTGAAGTGAGGACC | |
| *GmCSD6* | Forward: TGAAGGCTGTGGCAGTTCTT | |
|  | Reverse: CACCGTGCTCGTTGTTATTAGG | |
| *GAPDH* | Forward: GACTGGTATGGCATTCCGTGT | |
|  | Reverse: GCCCTCTGATTCCTCCTTGA | |
