# Supplementary material for: Genome-wide identification and characterization of the soybean SOD family during alkaline stress
Source: PeerJ. 2020 Feb 5;8:e8457. doi: 10.7717/peerj.8457 (PMC7007734; doi:10.7717/peerj.8457)
Supplement: Supplemental Information 9 [file peerj-08-8457-s009.docx]

**Table S2. Ka/Ks and divergence analysis of SOD paralogous in soybean**

| **Genes** | **Ka** | **Ks** | **Ka/Ks** | **Negative selection** | **Positive selection** |
| --- | --- | --- | --- | --- | --- |
| *GmMSD1/GmMSD2* | 0.0188 | 0.0134 | 1.4029 | no | yes |
| *GmFSD1/GmFSD2* | 0.0547 | 0.0516 | 1.0600 | no | yes |
| *GmFSD2/GmFSD3* | 0.1587 | 0.2076 | 0.7644 | yes | no |
| *GmFSD3/GmFSD5* | 0.0371 | 0.0147 | 2.523 | no | yes |
| *GmCSD1/GmCSD6* | 0.0581 | 0.0266 | 2.1842 | no | yes |
| *GmCSD2/GmCSD3* | 0.0538 | 0.0699 | 0.7696 | yes | no |
| *GmCSD2/GmCSD4* | 0.2253 | 0.2052 | 1.0979 | no | yes |
| *GmCSD3/GmCSD4* | 0.2131 | 0.1270 | 1.6779 | no | yes |
