# Supplementary figures and images for: Genome-wide identification and characterization of the soybean SOD family during alkaline stress
Source: PeerJ. 2020 Feb 5;8:e8457. doi: 10.7717/peerj.8457 (PMC7007734; doi:10.7717/peerj.8457)

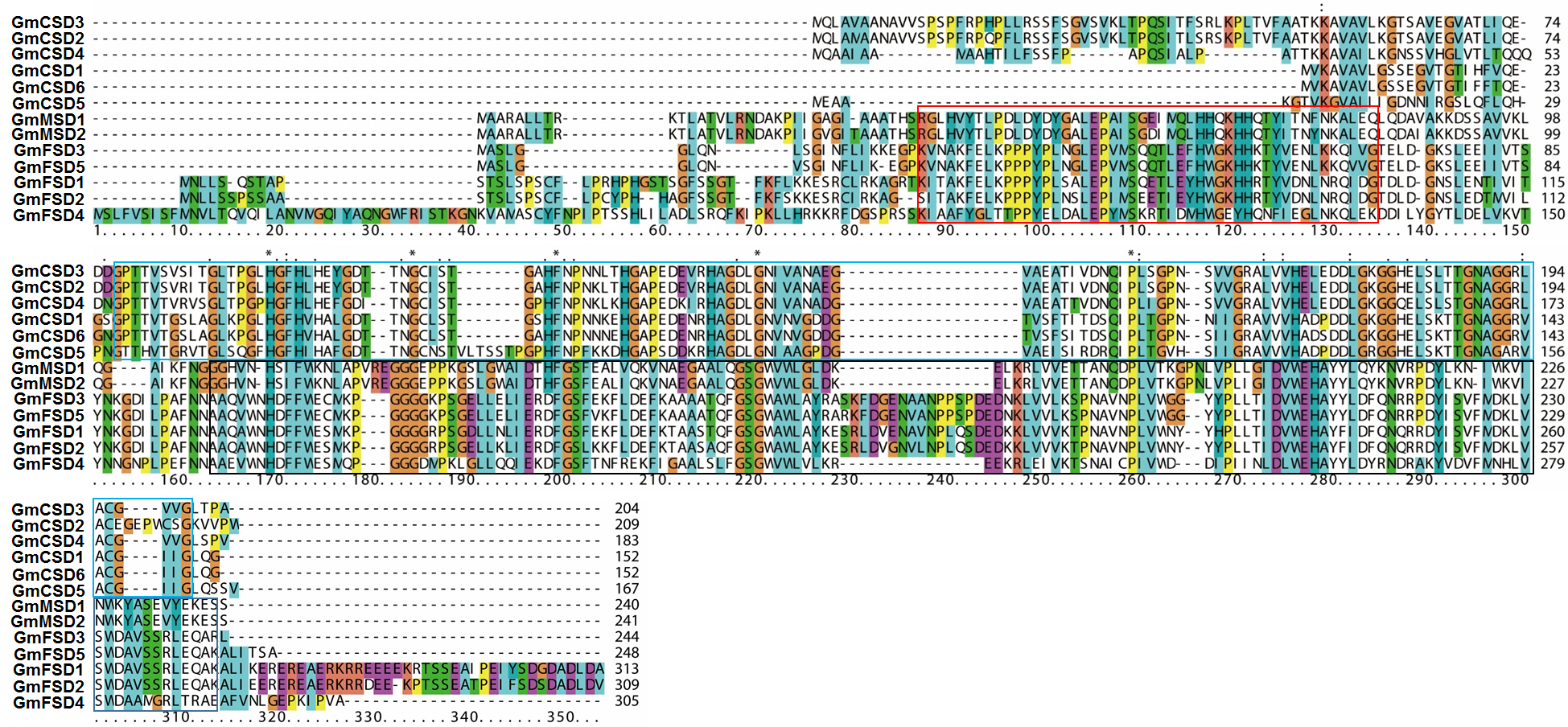

Supplement: Supplemental Information 11 — The copper/zinc SOD domain of Cu/ZnSODs subfamily members are marked with blue box. The alpha-hairpin domain and C-terminal domain of MnSODs and FeSODs subfamily members are marked with red and black boxes, respectively. [file peerj-08-8457-s011.jpg]

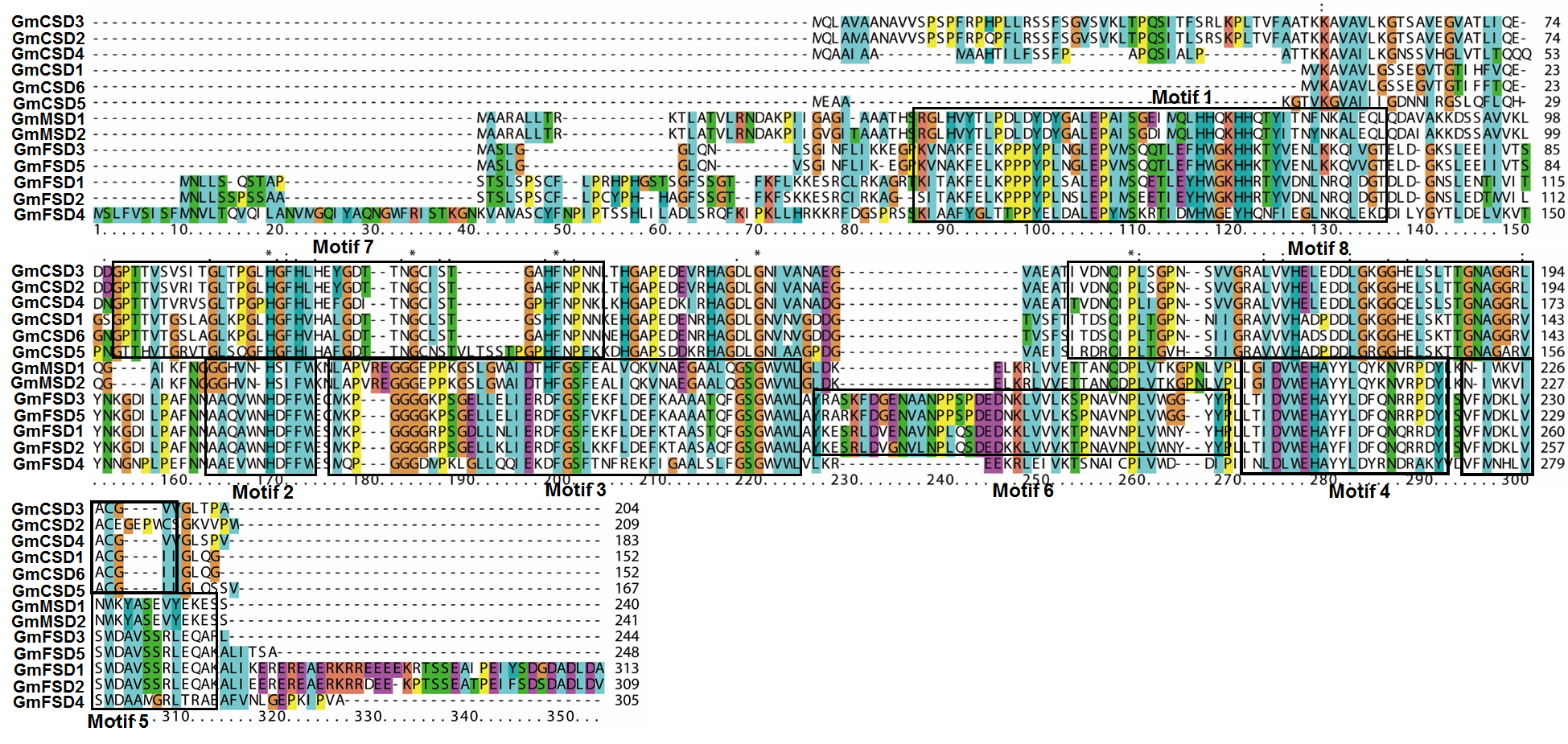

Supplement: Supplemental Information 12 — The motifs of SOD families in soybean are marked with black box. [file peerj-08-8457-s012.jpg]

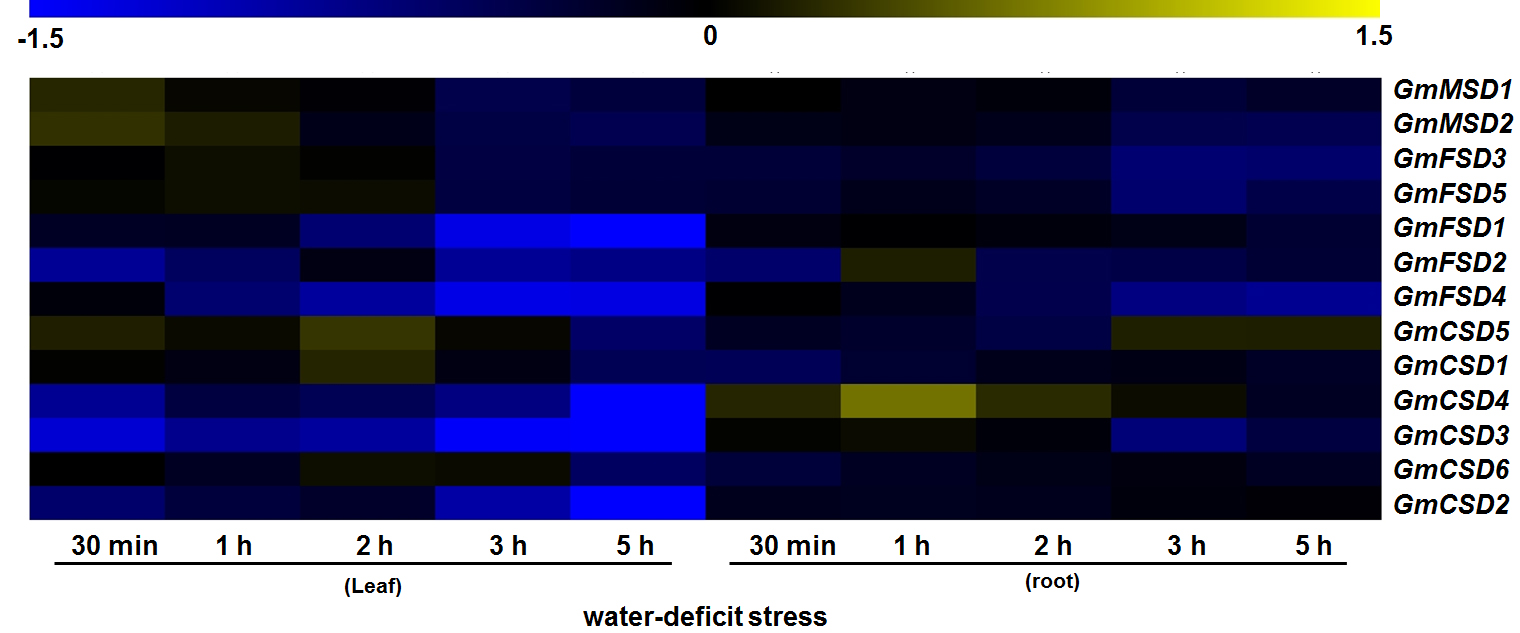

Supplement: Supplemental Information 13 — The color scale represents the expression values: blue indicates low levels and yellow represents high levels. [file peerj-08-8457-s013.jpg]
